# A 3D Collagen-Based Bioprinted Model to Study Osteosarcoma Invasiveness and Drug Response

**DOI:** 10.3390/polym14194070

**Published:** 2022-09-28

**Authors:** Evelin Pellegrini, Giovanna Desando, Mauro Petretta, Antonella Cellamare, Camilla Cristalli, Michela Pasello, Maria Cristina Manara, Brunella Grigolo, Katia Scotlandi

**Affiliations:** 1 Laboratory of Experimental Oncology, IRCCS Istituto Ortopedico Rizzoli, Via di Barbiano 1/10, 40136 Bologna, Italy; 2 Laboratory RAMSES, IRCCS Istituto Ortopedico Rizzoli, Via di Barbiano 1/10, 40136 Bologna, Italy; 3 REGENHU Ltd., Z.I. Le Vivier 22, 1690 Villaz-Saint-Pierre, Switzerland

**Keywords:** osteosarcoma, bioprinting, collagen-based hydrogels, cell proliferation, cisplatin resistance

## Abstract

The biological and therapeutic limits of traditional 2D culture models, which only partially mimic the complexity of cancer, have recently emerged. In this study, we used a 3D bioprinting platform to process a collagen-based hydrogel with embedded osteosarcoma (OS) cells. The human OS U-2 OS cell line and its resistant variant (U-2OS/CDDP 1 μg) were considered. The fabrication parameters were optimized to obtain 3D printed constructs with overall morphology and internal microarchitecture that accurately match the theoretical design, in a reproducible and stable process. The biocompatibility of the 3D bioprinting process and the chosen collagen bioink in supporting OS cell viability and metabolism was confirmed through multiple assays at short- (day 3) and long- (day 10) term follow-ups. In addition, we tested how the 3D collagen-based bioink affects the tumor cell invasive capabilities and chemosensitivity to cisplatin (CDDP). Overall, we developed a new 3D culture model of OS cells that is easy to set up, allows reproducible results, and better mirrors malignant features of OS than flat conditions, thus representing a promising tool for drug screening and OS cell biology research.

## 1. Introduction

Osteosarcoma (OS) is a highly aggressive bone malignancy that primarily affects adolescents and young adults. The standard-of-care regimen is based on local surgical control of the disease combined with high-dose chemotherapy. First-line conventional drugs include methotrexate, doxorubicin, cisplatin, and possibly ifosfamide (MAPI) or etoposide [1,2]. Despite the various attempts to ameliorate clinical outcomes, the survival rate has not improved over the past three decades; indeed, the 5-year survival rate for localized OS is still 60–70%, whereas survival for patients who do not respond to first line-therapy and develop metastases drops to 20–30% [1,3,4,5]. The occurrence of drug resistance [6,7] and development of metastasis (lung metastasis in 80% of the cases) [8] are strongly associated with poor prognosis, and represent critical hallmarks in OS progression [9,10]. In particular, chemoresistant and/or metastatic OS are heterogeneous, genetically complex, and therapeutically challenging because such cases cannot be treated by surgery and are refractory to conventional chemotherapy. Many new therapeutic options, including targeted therapy, immunotherapy, and intensive chemotherapy with peripheral blood stem cell reinfusion, are under investigation, but none have yet shown a major change in overall survival [11,12,13,14].

A better understanding of the mechanisms of drug resistance and metastasis formation is, therefore, mandatory to find new therapeutic targets. In addition, the clarification of the interactions between OS cells and the complex microenvironment of bone and bone marrow is highly required, since the tumor microenvironment plays a critical role in regulating cancer invasion and drug resistance [15]. In this respect, three-dimensional (3D) cell cultures offer the opportunity to simulate microenvironmental conditions, such as hypoxia and nutrient gradients, to co-culture two or more different cell types, and recapitulate native cell architecture and stiffness, a condition known to affect the cell behavior also in terms of gene expression profile and drug sensitivity. Moreover, 3D models allow to observe the invasive processes in real-time, interrogate the molecular pathways involved in cell migration and proliferation, and screen compounds with the potential anticancer activity in a condition that better represents in vivo tissues than conventional 2D cultures. Consequently, 3D cell cultures are increasingly becoming popular tools for basic research and drug screening. However, they do often rely on manual, multi-step fabrication techniques, which may limit the acquisition of repeatable and standardized results. In this respect, advanced bio-fabrication techniques, such as bioprinting, represent promising innovative approaches for developing physiologically relevant tissues or disease models. Through the simultaneous printing of cells, biomaterials, and bioactive cues with accurate spatial control, bioprinting allows the production of highly complex 3D microtissues, and enables the creation of models through an automated, repeatable approach, with the virtue of providing accurate control for in vitro biological and/or drug screening assays [16,17]. In the context of OS, 3D printing can produce novel bone tissue engineering scaffolds with customized shape, architecture, favorable macro-microstructure, mechanical strength, and cellular components [18].

In this study, we present an innovative preclinical 3D OS model through a 3D bioprinting platform using a natural derived, collagen-based bioink. Hydrogels have been widely used as scaffolds for tissue engineering because of their excellent biocompatibility. Among hydrogel materials (such as alginate, fibrinogen, gelatin, hyaluronic acid, and collagen), collagen was here favored to better mimic the natural bone mechanical environment. We introduced the 3D bioprinting process and the method of construction of the 3D tumor models, verified whether these scaffolds were biocompatible in maintaining cell viability, and tested their biological potential for evaluating cancer cell proliferation and spreading compared to 2D conditions. The results of biological characterization of cell proliferation, invasion, and chemosensitivity together with cell expression of matrix metalloproteinases (*MMPs*) and biomarkers of resistance to cisplatin (CDDP) for the printed 3D OS tumor models are shown.

## 2. Materials and Methods

### 2.1. OS Cell Lines

We used U-2 OS and its CDDP-resistant variant, U-2OS/CDDP 1 μg, to develop a simplified 3D OS model. The U-2OS/CDDP 1 μg cell line was established by exposing the CDDP-sensitive U-2 OS purchased from the American Type Culture Collection (ATCC) to step-by-step increases in CDDP (#P4394, Sigma Aldrich St Louis, MO, USA; concentrations as previously described [19]). Cell lines were tested for mycoplasma contamination every 3 months (#LT07-318, MycoAlert Mycoplasma Detection Kit, Lonza, Basel, Switzerland) and were authenticated (STR profiling) by analysis of the following loci: AMEL, D3S1358, TH01, D21S11, D18S51, D10S1248, D1S1656, D2S1338, D16S539, D22S1045, VWA, D8S1179, FGA, D2S441, D12S391, D19S433, and SE33 (last control July 2020; POWERPLEX ESX 17 Fast System, Promega, Madison, WI, USA). All cell lines were immediately amplified to constitute liquid nitrogen stocks and were never passaged for more than 1 month upon thawing. Cells were cultured with Iscove’s Modified Dulbecco’s Medium (#ECB2072L, IMDM, Euroclone, Milan, Italy) containing 10% heat-inactivated fetal bovine serum (FBS, #ECS0180L, Euroclone Milan, Italy), penicillin (100 units/mL), and streptomycin (100 μg/mL) (#ECB3001D, Euroclone Milan, Italy) maintained at 37 °C in a humidified 5% CO_2_ atmosphere.

### 2.2. Design and Fabrication of a 3D Osteosarcoma Model

The development of the 3D in-vitro model foresaw the design and fabrication of cell-laden 3D scaffolds using a bioprinting platform. For the first phase, a dedicated CAD software (BioCAD, RegenHU, CH) was used to design the 3D structures, characterized by a 10 × 10 mm square basis and a total height of 2 mm. A 0/90° infill pattern was selected as the internal microarchitecture, with a fiber diameter of 300 µm and pore size of 1700 µm. To guarantee an optimal adhesion within the different deposited layers, a layer height equal to 200 µm, approximately 70% of the fiber diameter, was selected, leading to the stacking of 10 layers to achieve the desired construct height. The design software enabled us to set the printing process to be automatically replicated within 6-well plates for higher throughput fabrication. The first phase of the fabrication of the 3D OS model included the preparation of the cell-laden bioink, according to the protocol provided by the manufacturer.

First, 1 mL of high-density collagen bioink (80 mg/mL Viscoll, Imtek, RU) was neutralized with a solution composed of 100 µL buffer (Imtek, RU) and 400 µL FBS. Preliminary analyses were carried out to test the biocompatibility of the buffer on OS cell lines through Trypan Blue solution (#T8154-100ML, Sigma-Aldrich St Louis, MO, USA) cell count at 1, 3, 6, and 24 h. U-2 OS and U-2OS/CDDP 1 μg cells were trypsinized, counted, and re-suspended at 2 × 10^6^ cells/mL in 300 µL of FBS. The suspension was then mixed with the neutralized collagen ink through a Luer-lock coupler (Sarstedt AG & Co. KG, Numbrecht, DE), performing 20 mixing iterations by gently and alternately pushing the two syringe plungers, as per the protocols provided by the manufacturer. Finally, the cell-laden bioink was loaded into the bioprinter cartridge.

The 3D bioprinting process was performed through a 3D Discovery platform (RegenHU, CH). High-precision microvalve-based dispensing technology, used in contact mode with a dedicated 300 µm needle, was chosen to maximize the printing fidelity of the low viscosity bioink. The printhead was kept at room temperature (RT) throughout the experiment, whereas the build zone temperature was set at 37 °C to favor physical gelation of the collagen bioink and improve the shape retention, as well as the stacking behavior, of the deposited fibers. The process was automatedly replicated in sterile ultra-low attachment 6 well-plates (#3471, Corning Inc., New York, NY, USA). Sterility was guaranteed by the printing platform being integrated in a class II laminar flow hood. As technical control, we used cell lines mixed with the collagen bioink, which did not undergo the printing process, to evaluate the effect of the procedure and the selected parameters on cell viability. Cell-laden hydrogels were kept in culture in the same experimental conditions reported above for up to 10 days by changing the culture media twice per week (Video S1). During the culture time, cells in hydrogels were observed through a phase-contrast microscope before all the scheduled assessments.

### 2.3. Analysis of Biological Parameters in 3D Conditions

#### 2.3.1. Cell Viability and Morphology

The viability of OS cells in the cell-laden bioinks was determined by using a Live/Dead^®^ cell viability kit (#L3224, Invitrogen, Waltham, MA, USA) following the manufacturer’s instructions. Briefly, constructs were rinsed with phosphate-buffered saline (PBS) (Sigma-Aldrich, St. Louis, MO, USA) and then incubated with calcein-AM and ethidium bromide solution (#L3224, Invitrogen, Waltham, MA, USA) for 30′ at 37 °C. After three washing steps with PBS, constructs were visualized with the epi-fluorescent microscope, Eclipse 90i (Nikon, Melville, NY, USA), using the fluorescein isothiocyanate (FITC) and tetramethyl rhodamine (TRITC) filters to evaluate the viable (green) and dead (red) cells, respectively. Beyond 2D microscopic acquisition, we performed Z-stack imaging in real-time from the top to the bottom for 3D reconstruction of some areas of constructs through Eclipse 90i software (Nikon, Melville, NY, USA). In parallel, viability and metabolic activities of OS cells in the 3D bioinks were assessed by Alamar Blue^TM^ Cell Viability Reagent (#DAL1100, Invitrogen, Thermo Scientific, Waltham, MA, USA) at day 3, 7, and 10. The 3D constructs were incubated with complete medium supplemented with 10% Alamar Blue^TM^ (Invitrogen, Thermo Scientific, Waltham, MA, USA) for 4 h a 37 °C in humidified 5% CO_2_. After the incubation, 100 µL of the solution was transferred to a 96-well plate, and the fluorescence signals were measured at an excitation length of 530–560 nm and an emission length of 590 nm by a Glomax Multi Detection System (Promega Italia S.r.l, Milano, Italy). A morphological evaluation of OS cells grown in the 3D bio-printed constructs was also performed at day 3, 7, and 10 after cell seeding. Three-dimensional constructs were embedded in the Tissue-Tek^®^ optimal cutting temperature (OCT) compound (#4583, Sakura Finetech, CA, USA), and then snap-frozen in liquid nitrogen. After cutting with the cryostat 12-μm thick sections, slides were fixed with paraformaldehyde solution 4% (#P6148, Sigma Aldrich St Louis, MO, USA), and then processed for Gill III Hematoxylin-Eosin (H/E) (#05-M06015, Bio-Optica, Milano, Italy) staining to evaluate the morphology of U-2 OS and U-2OS/CDDP 1 μg and cancer cell crosstalk with the collagen-based hydrogen at different time points.

#### 2.3.2. Cell Migration and Proliferation

Morphological evaluation of the invasive capabilities of OS cells in the bioprinted collagen scaffolds was performed after H/E staining of sections from 3D constructs embedded in OCT over 3–10 days. Evaluation of the expression of MMPs and the biomarker of chemoresistance was performed on day 10. The expression of Ki-67 was also determined as a measure of cell proliferation.

#### 2.3.3. Drug Response

Two 10^6^ U-2 OS or U-2OS/CDDP 1-ug cells were bio printed, cultured in hydrogels for 3 days, and checked for morphology and vitality before being exposed to different doses of CDDP (U-2 OS: 300 ng/mL, 1 μg/mL, and 3 μg/mL; U-2OS/CDDP 1 μg: 1 μg/mL, 3 μg/mL, and 10 μg/mL). Drug efficacy was evaluated over 3–10 days using either Live and Dead or Alamar Blue assays. IC_50_ (concentration required to inhibit cell proliferation by 50%) values were calculated from the linear transformation of dose–response curves.

### 2.4. Analysis of Biological Parameters in 2D Conditions

Cells were plated into 96-well plates (2000 cells/well) in standard medium and cultured for 24 h, before they were treated with CDDP for an additional 96 h, roughly equivalent to at least two doubling times of each cell line. Drug efficacy was assessed with the TACS^®^ MTT Cell Proliferation Assay kit (#4890-25-K, Trevigen, Inc., Gaithersburg, MD, USA) according to the manufacturer’s instructions.

For evaluating the expression of Ki-67, MMPs, and/or biomarkers of chemoresistance, 2 × 10^5^ cells were plated in 6-well dishes. At day 3 from cell seeding, when cells are in active growth, Ki-67 expression was assessed by immunofluorescence, and total RNA was extracted for gene evaluation by quantitative PCR (q-PCR).

### 2.5. Immunofluorescence

For 3D conditions, OS cell-laden bioinks were kept in culture for 3–10 days, and each sample was embedded in the OCT compound and snap-frozen. Slides were fixed in paraformaldehyde solution 4% (Kaltek srl, Padova, IT), and hydrated with Tris-buffered saline (TBS, #T5912, Sigma-Aldrich, St Louis, MO, USA) and the albumin solution 1% (#A4503, Sigma-Aldrich St Louis, MO, US) to block the non-specific antigenic sites for 30′ at RT. Then, samples were incubated with the mouse monoclonal anti-human antibodies, Ki-67 (clone UMAB107, #UM800033; Origene, Rockville, MD, USA; 10 µg/mL) or MMP-13 (Clone 87512, #MAB511, R&D Systems, CA, USA; 5 μg/mL). Following the washing steps in TBS, sections were incubated with the goat anti-mouse secondary antibody conjugated with FITC (#31569; Thermo Fisher Scientific, Waltham, MA, USA; 15 µg/mL) for 1 h at 4 °C. Negative controls were carried out either by avoiding the use of the primary antibodies or with isotype control. After three washing steps, samples were mounted with the anti-fading solution containing 4′,6-diamidine-2-phenylindole (DAPI, #D9542, Sigma St Louis, MO, USA) for nuclear staining. Section assessment was performed through the epi-fluorescent microscope, Eclipse 90i (Nikon). Ten microscopic fields (40× magnification) were used to perform a semi-quantitative analysis of immunofluorescence for Ki-67 and MMP-13 markers. Technical triplicates were used for each time point.

For 2D conditions, cells were fixed with 4% paraformaldehyde solution, and then immunoassayed as for 3D sections.

### 2.6. RNA Extraction and Quantitative PCR

The total RNA from OS cells constructs was extracted with TRIzol Reagent (#15596018; Thermo Fisher Scientific—Life Technologies, Grand Island, NY, USA) following the manufacturer’s instructions. RNA quality and quantity were assessed by NanoDrop analysis (NanoDrop ND1000, Thermo Scientific, MA, USA). The total RNA from each sample was reverse-transcribed into complementary DNA (cDNA) using a High-Capacity cDNA Reverse Transcription Kit (#4368813; Thermo Fisher Scientific—Applied Biosystems, Waltham, MA, USA) according to the manufacturer’s protocols. Quantitative reverse transcriptase-polymerase chain reaction (qPCR) was performed on a ViiATM 7 System (Applied Biosystems, Waltham, MA, USA) using TaqMan Universal PCR Master Mix (#4304437, Thermo Fisher Scientific-Applied Biosystems) and SYBR Green PCR Master Mix (#4312704, Thermo Fisher Scientific—Applied Biosystem, Waltham, MA, USA). Predesigned TaqMan assays (#4351370, Thermo Fisher Scientific—Applied Biosystems, Waltham, MA, USA) were used for the following genes: *GAPDH* (Hs99999905_m1), *GSTP1* (Hs00168310_m1), *XPA* (Hs00166045_m1), *ERCC1* (Hs01012158_m1), *ERCC2* (Hs00361161_m1), *ERCC5* (Hs01557031_m1), *ERCC4* (Hs00193342_m1), *MMP-1* (Hs00899658_m1), *MMP-9* (Hs00234579_m1). We used the following primers for *MMP-13* (forward, 5′-TCA CGA TGG CAT TGC T-3′ and reverse 5′-GCC GGT GTA GGT GTA GA-3′) and *GAPDH* (forward, 5′- GAAGGTGAAGGTCGGAGTC-3′ and reverse, 5′- GAAGATGGCGATGGGATTTC-3′).

Relative quantification was performed using the 2^−ΔΔCT^ method [20]. Results were normalized to the level of the housekeeping gene, *GAPDH*, and were expressed as relative quantification (RQ; 2^−ΔΔCt^).

### 2.7. Statistical Analysis

All statistical analyses were performed using Prism version 7.0 (GraphPad Software, La Jolla, CA, USA). IC_50_ (concentration required to inhibit cell proliferation by 50%) values were calculated from the linear transformation of dose–response curves. IC_50_ values were calculated by fitting data into dose–response curves (inhibitor vs. response) by nonlinear regression with a variable slope. Comparisons between two groups were evaluated with two-tailed Student’s *t*-tests. Experimental data including more than 2 groups were analyzed using one-way or two-way ANOVA. The data were considered statistically significant at *p* < 0.05.

## 3. Results

### 3.1. Morphological Properties of the 3D OS Model

**Figure 1:**
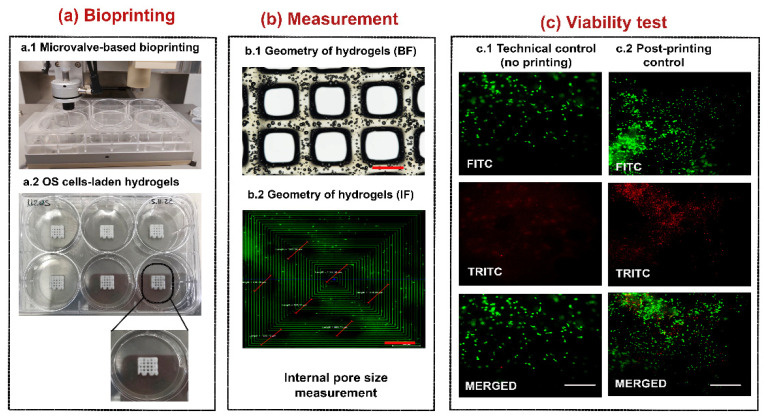
Schematic representation of the workflow of 3D bioprinting of OS cells. From left to right panels: (**a**) the bioprinting process, (**b**) measurement assessments, and (**c**) viability analyses. (**a.1**) The bioprinting process was performed with microvalve-based dispensing of the U-2 OS cell-laden bioink in ultra-low attachment 6-well plates. (**a.2**). Representative image of fabricated 3D OS models with high magnification (dashed lines) of a construct. (**b**). Measurement of pores within the representative micrograph of a 3D scaffold under bright-field (BF) (**b.1**) and fluorescence (IF) (**b.2**) microscopy (4× magnification; red scale bar at the bottom of b.1 and b.2 images: 1000 µm) for measurement assessments. The thinner red lines within the b.2 image represent pore measurements. (**c**)**.** Representative images of technical (no bioprinting) (**c.1**) and post-printing (**c.2**) controls were captured following Live (green staining) and Dead (red staining) assay; white scale bar at the bottom of merged images (**c**): 100 µm.

Among collagen bioink formulations available on the market, we used one of the highest concentrations (80 mg/mL in acidic form) for setting our experimental conditions to overcome potential issues related to low mechanical properties, and improve printability and shape retention [21]. A schematic of the inkjet printer setup with the different elements needed for this novel protocol is shown in Figure 1.

The U-2 OS cell line and its resistant variant, U-2OS/CDDP 1 μg, were bio-printed in a high-density collagen bioink employing microvalve-based dispensing technology. The fabrication parameters were optimized to obtain 3D-printed constructs with overall morphology and internal microarchitecture accurately matching the theoretical design, in a reproducible and stable process. They are reported in Table 1.

Bright-field microscopy observation enabled us to test the correspondence of average fiber and pore size diameters to select the bioprinting parameters set. The fabricated construct presented a fiber diameter of 341.89 ± 20.12 µm and pore size of 1701.78 ± 53.58 µm. The vital dye for cell counting did not reveal any toxicity for OS cell lines after their mixture with the collagen bioink (cell viability: 99.5%). Similarly, technical controls with no printing or post-printing confirmed the absence of cell toxicity following Live (green staining) and Dead (red staining) assay (Figure 1).

### 3.2. Assessment of Cell Viability, Proliferation, and Migration of OS Cells

**Figure 2:**
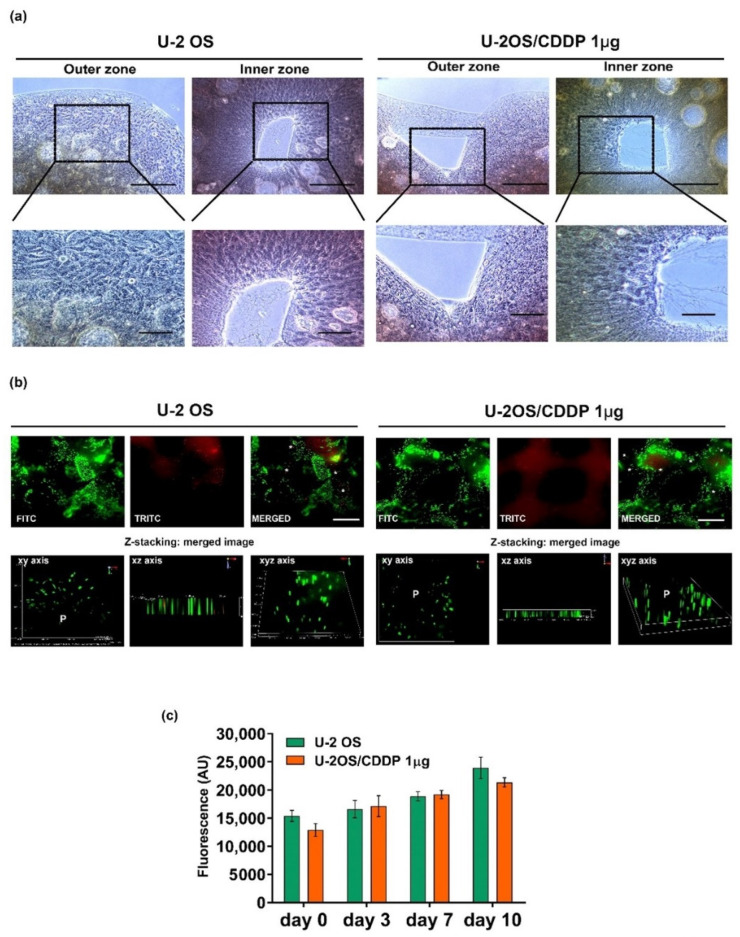
(**a**) Representative images of U-2 OS and U-2OS/CDDP 1 μg on collagen hydrogels captured with the phase-contrast microscope at day 7. Scale bar = 100 μm. (**b**) Representative fluorescent micrographs of live (green) and dead (red) bio-printed U-2 OS and U-2OS/CDDP 1 μg cells on collagen hydrogels at day 7 through Live and Dead assay. Epi-fluorescence microscope, equipped with a 10/0.25 water immersion objective lens and FITC and TRITC filters, was used. Asterisks: dead cells. Scale bar = 100 μm. Z-stacking, which provides three-dimensional data (xy axis, xz axis, xyz axis) of multiple focal planes (P), is shown. (**c**) Metabolic activity of U-2 OS and U-2OS/CDDP 1 μg cells on collagen hydrogels was assessed over the time by Alamar Blue. Data are reported as mean ± standard errors.

The cytocompatibility of the collagen hydrogel post-printing process (95% ± 4%) was then tested at early- (day 3) and long- (day 7 or 10) term follow-ups. We monitored the OS cell behavior in the 3D collagen hydrogel through bright-field microscopy daily, whereas Live and Dead and/or Alamar Blue assays were performed at day 3, 7, or 10. The phase-contrast microscope showed an increased number of cells from day 3 to day 10 for both cell lines, with a uniform cell distribution along with the collagen hydrogels. Representative images at day 10 showed that OS cells were indeed distributed in the inner region of the collagen hydrogel near the pore areas and on the edge of the collagen (Figure 2a). The Live and Dead assay showed good viability (about 90% ± 5%) for both cell lines at all the time points, with similar values to the technical controls (Figure 2b). In addition, the Alamar Blue test confirmed the increased metabolic activity over the time of OS cells bio-printed in the collagen hydrogel (Figure 2c).

**Figure 3:**
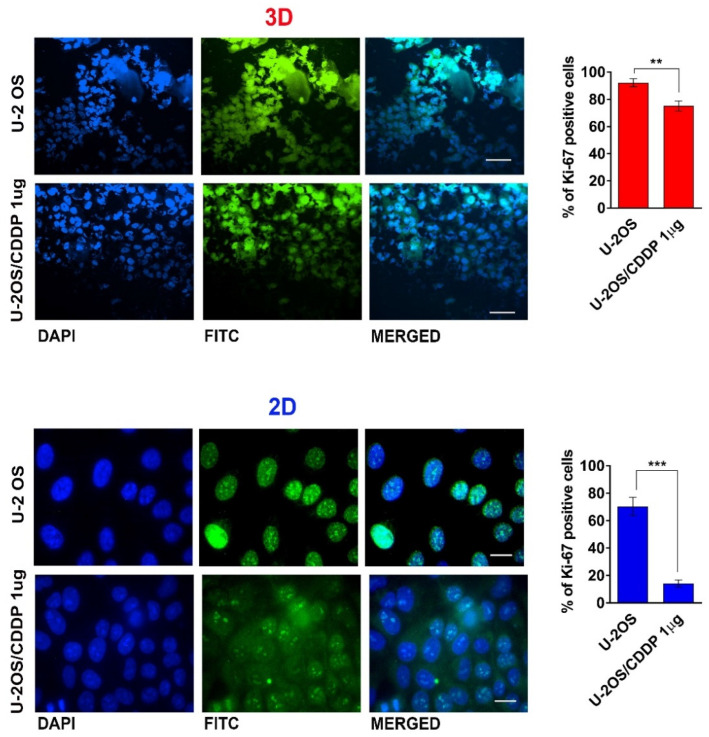
Representative fluorescent images and percentage of Ki-67 positivity in U-2 OS and U-2OS/CDDP 1 μg cultured in the 3D or in the 2D culture models. From left to right, the panels in each row show fluorescence from DAPI (nuclei—blue staining), FITC (positivity for Ki-67), and merged images. 3D condition: magnification = X400; scale bar= 40 μm. 2D condition: magnification = ×600; scale bar = 20 μm. Graphical representation of the percentage of positivity for Ki-67 in each condition is also shown. A two-tailed Student’s *t*-test was used for statistical analysis. ** = *p* < 0.01; *** = *p* < 0.001.

These findings indicate that the experimental conditions are well-settled to maintain sufficient gas exchange and diffusion of nutrients, but limit waste accumulation, thus sustaining the viability and proliferative/metabolic activity of OS cells. The maintenance of the proliferation rate was confirmed through the immunostaining for Ki-67 (Figure 3). We compared the proliferation rate of OS cells in standard 2D vs. 3D culture conditions, and, interestingly, we observed a striking difference in the proliferation of the resistant cells. In fact, though, in 2D flat cultures, the U-2OS/CDDP 1 μg resistant cells showed a very low proliferative rate (14%), the positivity to Ki-67 increases by around five times, achieving the percentage of 75% in 3D cultures.

**Figure 4:**
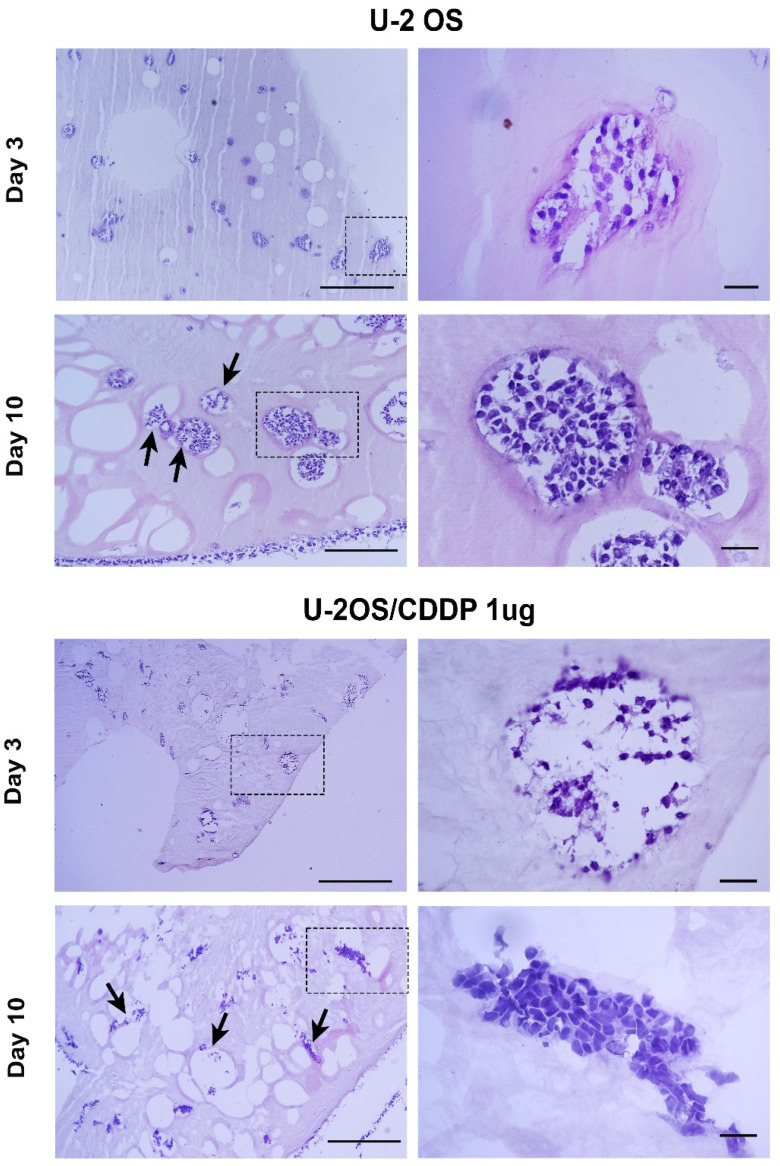
Representative micrographs of Hematoxylin/Eosin of U-2 OS and U2-OS/CDDP 1 μg-laden collagen hydrogels on day 3 and 10. The panels in each row show the section at low magnification (**left**) (scale bar, 200 μm) and the details marked with the dashed rectangle at high magnification (**right**) (scale bar = 20 μm). Arrows indicate the lacunae with invading OS cells.

We also tested the feasibility of collagen hydrogel to sustain the invasion capability of OS cell lines. Both cell lines showed cell clusters within the collagen hydrogels from day 3 to day 10; the dimension of cell clusters increased together with matrix degradation, as indicated by the presence of a higher number of empty, larger lacunae (Figure 4).

Cancer cells exploit these lacunae to migrate inwards the scaffold, generating spheroids after colonizing and proliferating within the small lacunae. U-2OS/CDDP 1 μg cells form longer cell clusters compared to U-2 OS cells, a feature that has been previously reported for more malignant cells [22]. We hypothesize that such lacunae are actively produced by the entrapped cells, and not created during the bio-fabrication process, since their number increased over the time. To test this hypothesis, the expression of the matrix metalloproteinases (MMPs)-1, -9 and -13, members of a family of zinc-dependent endoproteases, was also considered. These proteases are responsible for degrading the extracellular matrix (ECM) by breaking down various proteins in its structure and for promoting a wide spectrum of processes, including cell proliferation and migration/metastasis [23]

**Figure 5:**
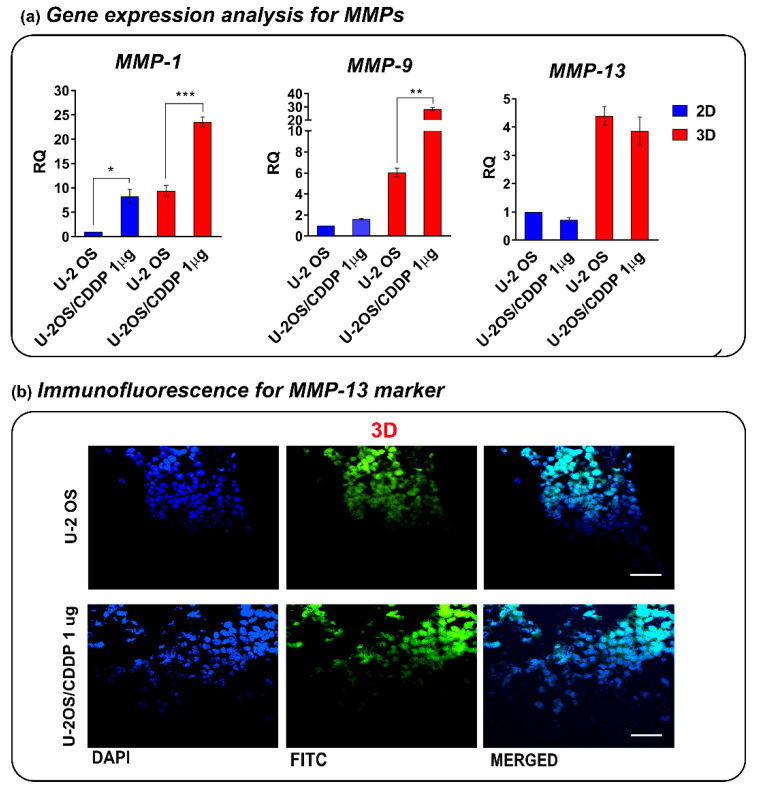
(**a**) Relative expression *of MMP-1*, *MMP-9*, and *MMP-13* in U-2 OS cells or chemoresistant variants in the 3D or 2D culture conditions by qPCR. The data are shown as the mean ± SE of two independent biological experiments performed in duplicate. RQ, relative quantification calculated as 2^−ΔΔCt^ (* *p* < 0.05, ** *p* < 0.01, *** *p* < 0.001, Student’s *t*-test). (**b**) Representative fluorescent micrographs for MMP-13 of U-2 OS and U-2OS/CDDP 1 μg cultured in the 3D hydrogels. From left to right, the panels in each row show fluorescence from DAPI (nuclei—blue staining), FITC (positivity for MMP-13), and merged images. Scale bar = 40 μm.

In keeping with previous evidence [24], the gene expression of *MMP-1* (also named collagenase-1) was strongly increased in the chemoresistant cells compared to parental cells, either in standard 2D or 3D conditions (Figure 5a). In addition, U-2OS/CDDP 1 μg cells also expressed a higher level of MMP-9 (also named gelatinase B) in 3D, in line with the presence of more abundant lacunae and cell infiltration (Figure 4). The gene expression of MMP-13 (also named collagenase 3) was enhanced in both OS cell lines following their culture in 3D conditions, although the general level of expression was lower than MMP-1 and -9. No significant difference for MMP-13 was found between the chemoresistant variant and parental OS cells in both 2D and 3D conditions. Of note, the expression of all the three MMPs is generally enhanced in 3D compared to the planar condition in the two cell lines. The expression of MMP-13 in OS cells when cultured in 3D hydrogels was also confirmed by immunofluorescence (Figure 5b). Conversely, the cell-free construct in culture did not alter the scaffold structure over time, confirming the role of MMPs in construct remodeling (Figure S1).

### 3.3. Drug Response

**Figure 6:**
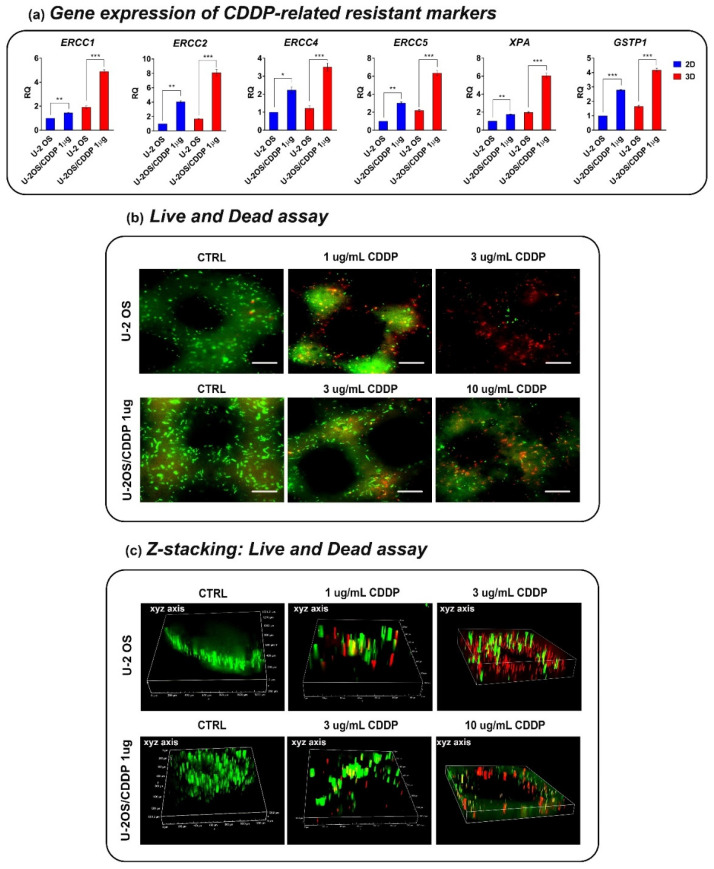
(**a**) Relative expression of *XPA*, NER gene *ERCC* excision repair (*ERCC*)-1, -2, -4, -5, *GSTP1* genes in U-2 OS or U-2OS/CDDP 1 µg in 3D versus standard 2D conditions by qPCR. The data are shown as the mean ± SE of two independent biological experiments performed in duplicate. RQ, relative quantification, calculated as 2^−ΔΔCt^ (* *p* < 0.05, ** *p* < 0.01, *** *p* < 0.001, Student’s *t* test). (**b**) Representative fluorescent micrographs of live (green) and dead (red) bio-printed U-2 OS and U-2OS/CDDP 1 μg cells after exposure to CDDP. Epifluorescence microscope, equipped with a 10/0.25 water immersion objective lens and FITC and TRITC filters, was used. Scale bar = 100 μm. (**c**) Representative Z-stack fluorescent images of Live and Dead assay in U-2 OS or U-2OS/CDDP 1 µg. Z-stacking provides three-dimensional data (xy axis, xz axis, xyz axis) of multiple focal planes, is shown.

**Figure 7:**
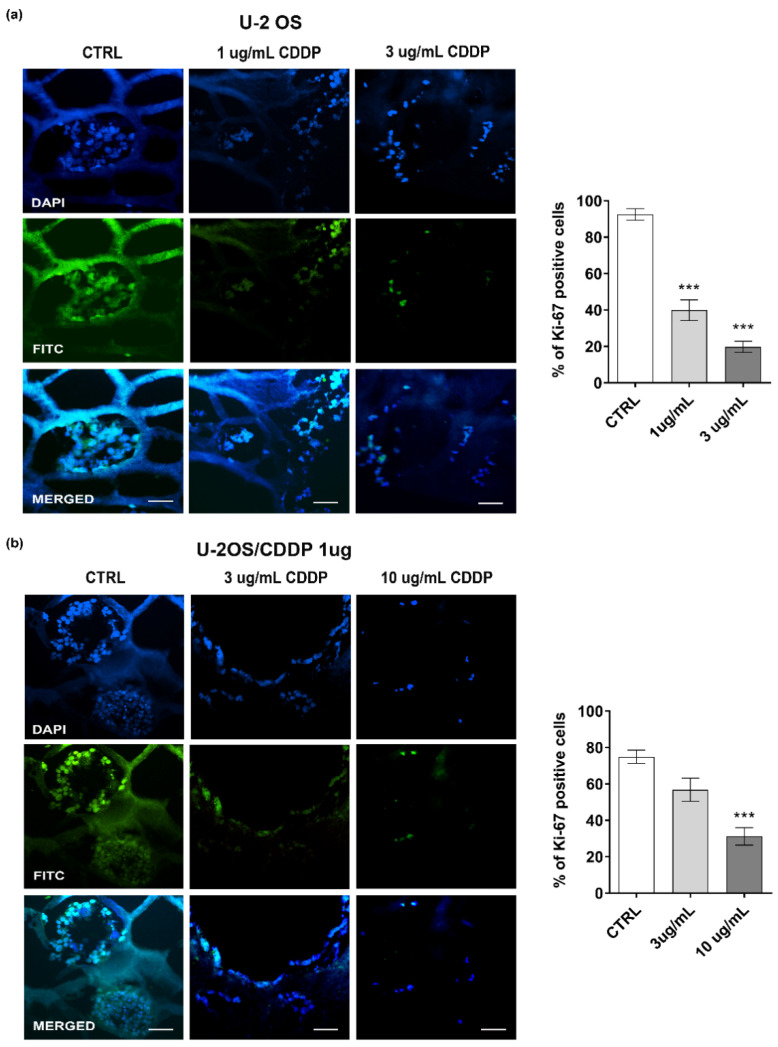
Representative fluorescent images and percentage of Ki-67 positivity in U-2 OS (**a**) and U-2OS/CDDP 1 μg (**b**) cultured in the 3D cultures and exposed to CDDP for 10 days. From left to right, the panels in each row show fluorescence from DAPI (nuclei—blue staining), FITC (positivity for Ki-67), and merged images. Scale bar = 40 μm. Graphical representation of the percentage of positivity for Ki-67 in each condition is also shown. A one-way ANOVA test was used for statistical analysis. *** = *p* < 0.001.

**Figure 8:**
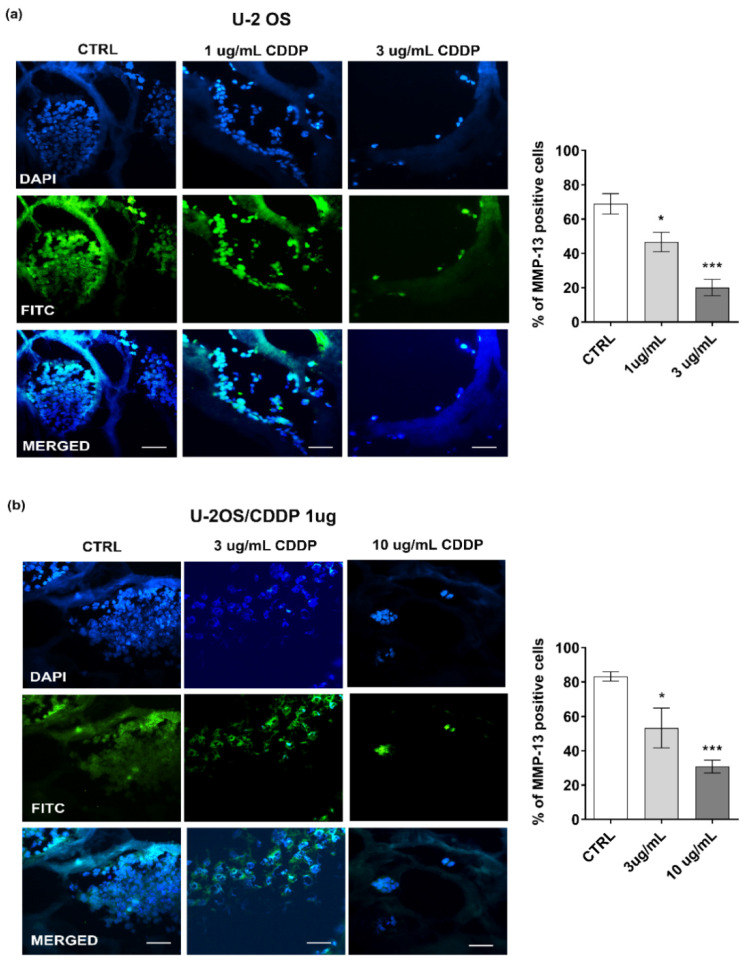
Representative fluorescent images and percentage of MMP-13 positivity in U-2 OS (**a**) and U-2OS/CDDP 1 μg (**b**) cultured in the 3D cultures and exposed to CDDP for 10 days. From left to right, the panels in each row show fluorescence from DAPI (nuclei—blue staining), FITC (positivity for MMP-13), and merged images. Scale bar = 20 μm. Graphical representation of the percentage of positivity for MMP-13 in each condition is also shown. A one-way ANOVA test was used for statistical analysis. * = *p* < 0.05; *** = *p* < 0.001.

We then assessed the chemosensitivity of OS cells in the 3D collagen hydrogel systems, and whether the 3D condition may influence drug resistance. As a first step, we verified the expression of several genes that were shown to be associated with resistance to CDDP (Figure 6a) [19]. In a drug-free environment, we confirmed that the expression of *XPA*, *ERCC1*, *ERCC2*, *ERCC4*, *ERCC5*, and *GSTP1* was higher in the chemoresistant variant than in parental cells. The 3D condition contributed to enhance the differences between the resistant U-2OS/CDDP 1 μg and U-2 OS cells, and, overall, it led to a higher expression of these markers compared to the flat condition both in parental and chemoresistant variant cells. In keeping with the highest expression of these biomarkers, the parental U-2 OS cells were less sensitive to CDDP in 3D versus standard conditions (IC_50_ value is 1.5 μM (95% confidence interval: 1.34 μM to 1.69 μM) vs. 2.7 μM (confidence interval: 2.67 μM to 3.15 μM); 1.8-fold higher in 3D vs. 2D) (Figure S2). The Live and Dead test (Figure 6b) and the expression of Ki-67 (Figure 7) also reported a CDDP dose-dependent reduction in the vitality and proliferation of U-2 OS cells or U-2OS/CDPP 1 μg cells after cell exposure to the chemotherapeutic agent. Moreover, the treatment with CDDP reduced the expression of MMP-13 in a dose-dependent manner (Figure 8).

## 4. Discussion

Three-dimensional cultures of cancer cells enable better mimicking of physiological conditions than traditional monolayer 2D cultures and are becoming a valuable tool in cancer cell biology research and drug screening. During recent years, different strategies have been exploited to study the behavior of cancer cells in 3D milieus thanks to the employment of advanced biotechnologies, different kinds of scaffolds, and the most sophisticated culture approaches [25]. According to the technology used, 3D cancer models include a variety of systems, such as scaffold-free systems, scaffold-based systems, hydrogel-based models, bioreactor-based models, microcarrier-based models, and cancer-on-a-chip [26]. In addition, bioprinting has been recently proposed to develop 3D models that combine cells and biomaterials (bioink) to obtain tissue-like structures by means of a layer-by-layer deposition [27]. In this study, we simultaneously printed OS cells (U-2 OS or its CDDP-resistant variant) together with a collagen bioink to develop collagen-based hydrogel constructs aimed at creating OS tumor models that better mimicked the features of the native microenvironment, and we studied the 3D tumor characteristics.

Cell survival rate is one of the key factors to consider while applying 3D cell printing technology in the construction of tissue-like models. Cells are subjected to mechanical forces during the 3D extrusion cell printing process, and it is well known that increased mechanical forces can cause cellular damage, reducing cell survival rate. Thus, one of the big challenges for bioprinting is to simultaneously print cells and the material (bioink) without affecting the cell viability. Here, we could confirm that the collagen-based hydrogels and the printing conditions used to model the bone extracellular matrix [28] preserve the viability and metabolic activity of OS cells. The chemical composition, relative abundance, and spatial organization of ECM constituents confer each tissue type with unique physical and biochemical properties (e.g., the rigidity of the matrix or its porosity) that may affect cell behavior. Collagen is widely used for engineering hard tissues, such as bone, because of its strength and toughness [29]. In addition, beyond mechanical properties, collagen fibers self-organize into 3D networks and can mimic the native matrix features, providing stimuli for cell adhesion, biodegradation sequences, and cues for supporting cell survival, growth, and migration [30]. The polymerization properties of collagen are highly dependent on its concentration, temperature, pH, ionic forces, and level of cross-linking [31]. Therefore, by properly tuning the polymerization parameters, we can change the mechanical properties of the scaffolds. In addition, in the design of the pore size, the physical hydrogel long-term swelling behavior and the need for higher porosity values should be considered to guarantee proper nutrient transport properties during culture conditions [32].

Herein, we present a novel 3D system in which all these parameters have been optimized to reproduce the mechanical constrain sensed by cells in primary tumors, and that successfully sustained OS cell proliferation and migration.

Cell migration is crucial for metastasis, the most relevant clinical challenge for oncologists treating OS patients. Cell assays based on 2D cellular models, such as wound healing or scratch-based assays, are widely used for migration research, but these systems cannot faithfully recapitulate the molecular and biomechanical complexity of in vivo environments. Indeed, 2D models are not able to replicate some specific features of the tumor environment, such as the cell’s spatial confinement, or cell–cell and cell–matrix interactions that affect the proliferation or the response to migration stimuli. Several MMP family members are among the main contributors of the degradation of ECM during tumor cell invasion [33].

In our experimental conditions, we found that either sensitive or resistant OS cells express higher levels of MMP-1, -9, and -13 in 3D compared to flat conditions. This discrepant behavior was particularly evident for the chemo-resistant U-2OS/CDDP 1 μg cells, which, in planar cultures, were less aggressive either in terms of invasion/migration or proliferation in comparison with parental cells, but that regained their aggressiveness when printed in collagen hydrogels.

Findings from the proliferation and migration assays, indeed, indicate that the collagen-based hydrogel is a suitable environment to preserve the tumor aggressiveness of CDDP-resistant OS cells. We can speculate that collagen may influence the proliferation of resistant cell more than that of sensitive ones because these cells have differential expression of collagen receptors on their surface. Cellular behavior is controlled by cell signal transduction pathways. Additionally, integrins (typical adhesion molecules in cancer cells), TGFβ receptors, and collagens closely associate with discoidin domain receptors (DDRs), a subfamily of tyrosine kinases that can be phosphorylated by collagen ligation, leading to the activation of AKT/PI3K signaling, mitogen-activated protein kinase (MAPK)/ERK signaling, and Rho family signaling [34], which regulate various functions, including cell proliferation and migration. In addition, cancer cells can also influence the expression and production of collagens, and their remodeling. For example, proteolysis of collagen is thought to be crucial for the active movement of tumor cells, as it opens migratory tracks and reduces mechanical stress on the migrating cells. In addition, the remodeling of collagens may be essential for releasing soluble growth factors, such as the liberation of insulin-like growth factors that were reported to sustain OS cell proliferation [30].

Thus, the highest expression of metalloproteinases that is induced by the 3D condition may explain the discrepancy in the positivity of Ki-67 that we observed for resistant cells between 3D and 2D cultures. Conversely, maintaining the cell-free construct in culture did not alter its structure over time. This indicates, on one side, the stability of the printed constructs, and, on the other side, gave evidence of the role exerted by MMPs in remodeling the collagen matrix after OS cell embedding.

Changes in the tumor microenvironment also affect drug sensitivity [35]. In this study, we demonstrated that the 3D condition enhanced the expression of genes implicated in the repair of CDDP-induced DNA damages [19,36] before OS cells were exposed to any chemotherapeutic drug, and decreased sensitivity to chemotherapeutic agents. It is widely described that collagen plays an important role in therapy resistance [34]. In addition, different collagen types may induce distinct treatment resistances. For example, in ER-positive cancer cells, COLI induced resistance to drugs, such as cisplatin and mitoxantrone, by activating β1 integrin followed by the FAK/PI3K/AKT pathway, whereas in triple-negative cancer cells, the MAPK pathway was found to be involved [37]. COL11A1 induced chemoresistance and exerted anti-apoptotic effects in ovarian cancer cells by mediating the transcriptional activation of NF-κB to upregulate the Twist family [38]. In our cellular model, the presence of collagen type I induced a strong expression of genes involved in DNA repair-related factors belonging to the nucleotide excision repair (NER) or base excision repair (BER) pathways and of the glutathione S-transferase P1 (GSTP1) either in sensitive or resistant cells. As proof-of-principle, we chose CDDP because it is a DNA-damaging agent currently used in the treatment of OS. Previous studies have clearly shown that overexpression of these genes negatively impact the OS cell responsiveness to CDDP-based treatments, as well as patient outcome [19,36]. Here, we show that the 3D hydrogel provided a better structural support than 2D conditions in fostering gene expression of DNA repair enzymes in sensitive and resistant OS cells. Overall, our data indicate that collagen-based hydrogels are suitable systems to study drug efficacy. These scaffolds provide reliable and reproducible results when used to measure the chemosensitivity of cells, and their use should be extended to other chemotherapeutics or targeted agents.

## 5. Conclusions

We describe a novel collagen-based hydrogel that can be used in modeling the biological features of OS cells and their chemosensitivity. This 3D culture model resembles in vivo conditions, it is easy to be set up, allows reproducible results, and better mirrors the malignant phenotype than flat conditions, making it a promising tool for drug screening and OS cell biology research. In perspective, this 3D bio-printed model might be empowered with normal cell components, such as stromal or immune cells, to allow more appropriate evaluation of immunotherapy or targeted therapy efficacy. The availability of reliable models is particularly relevant for rare tumors, for which in vitro prioritization of effective agents is mandatory to optimize the design of innovative clinical trials.

## Figures and Tables

**Table 1:** List of optimized printing parameters for developing the 3D OS model.

Needle Diameter	300 µm
Pressure	0.8 Bar
Valve opening time	280 µs
Dosing Distance	0.1 mm
Printing speed	8 mm/s
Cartridge Temperature	RT
Build zone (well-plate) temperature	37 °C

## Data Availability

The data presented in this study are available on request from the corresponding author.

## Supplementary Materials

The following supporting information can be downloaded at: https://www.mdpi.com/article/10.3390/polym14194070/s1. Figure S1. Histological assessment of OS-cell-laden collagen-based hydrogel: scan-large images; Figure S2. IC_50_ of U-2 OS or U-2OS/CDDP 1 μg in 2D versus 3D OS models; Video S1. Bioprinting experiment preparation.
