# Supplementary figures and images for: A 3D Collagen-Based Bioprinted Model to Study Osteosarcoma Invasiveness and Drug Response
Source: Polymers (Basel). 2022 Sep 28;14(19):4070. doi: 10.3390/polym14194070 (PMC9571197; doi:10.3390/polym14194070)

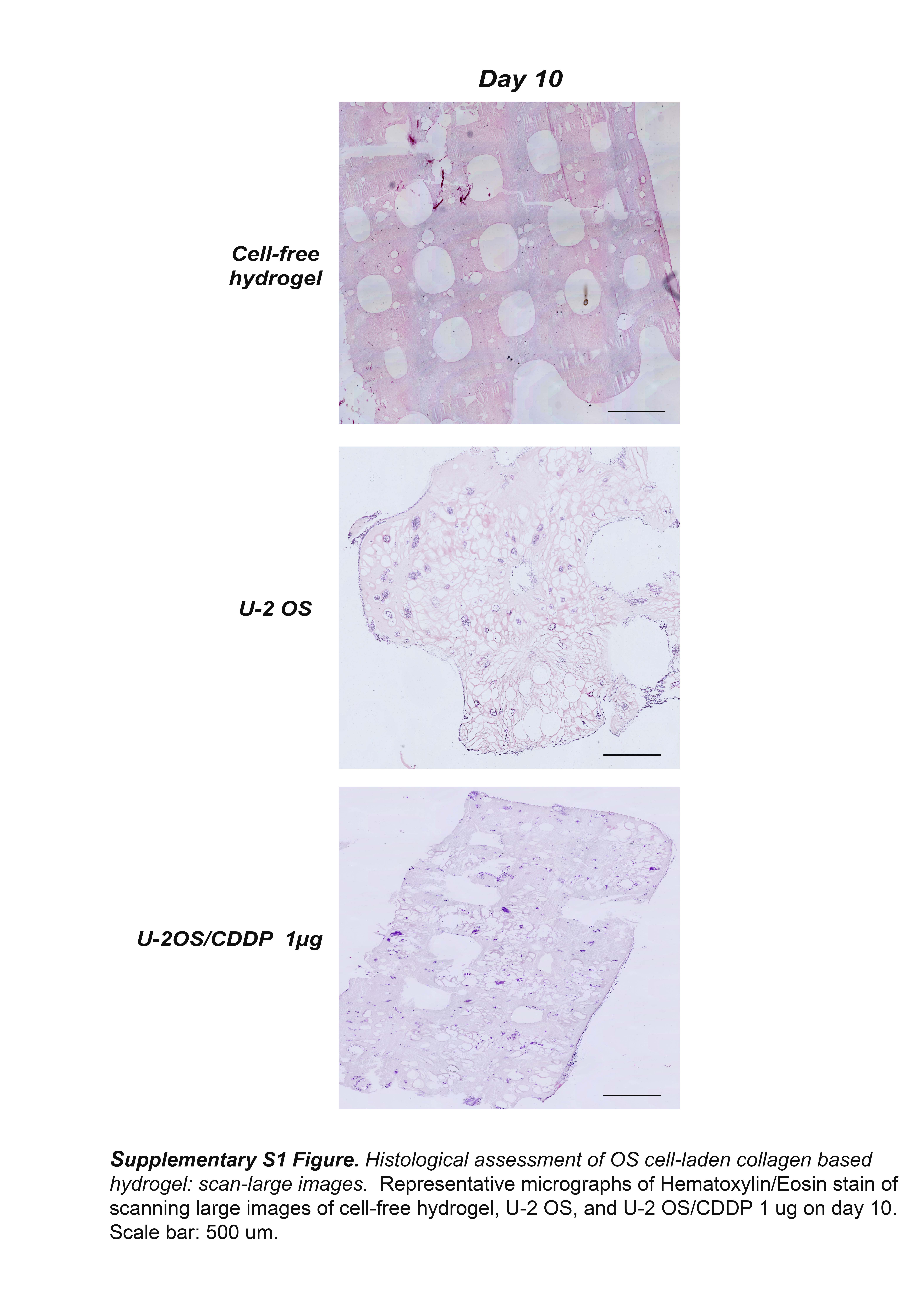

Supplement: Supplementary file 1 [file polymers-14-04070-s001.zip › S1 supplemetary figure rev.jpg]

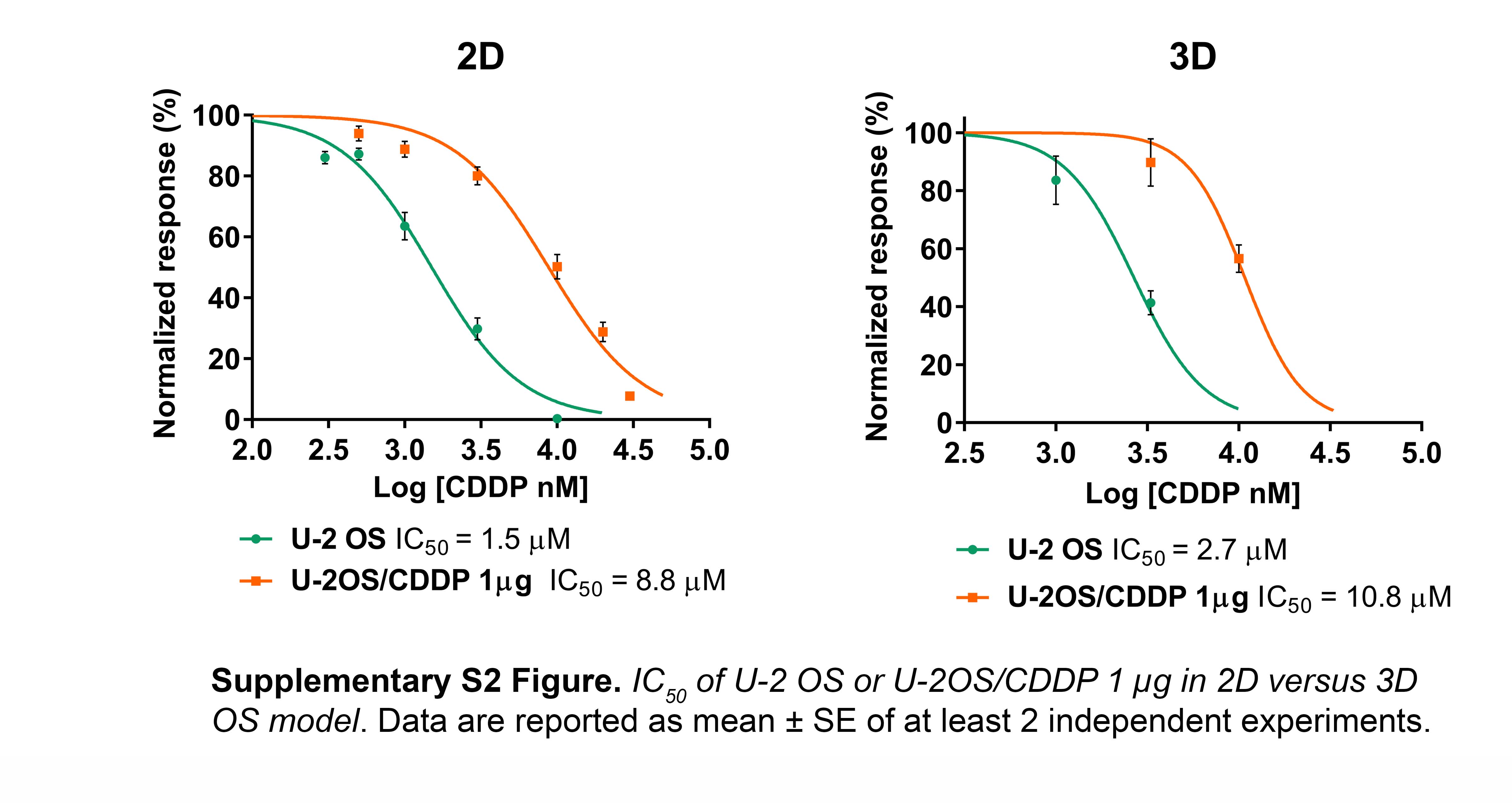

Supplement: Supplementary file 1 [file polymers-14-04070-s001.zip › S2 supplementary figure rev.jpg]
